# In Memoriam—Samuel Hahnemann Jackson, M. D.

**Published:** 1897-04

**Authors:** John Storer


### IN MEMORIAM.—SAMUEL HAHNEMANN JACKSON, M. D.

Samuel Hahnemann Jackson, M. D., died February 28th, 1897, of Bright's disease, at his home in Jamaica Plain, Mass., at the age of fifty-three. He had been in poor health for a number of years, but not until last November was he obliged to relinquish his large practice. Dr. Jackson was a successful, popular physician, and had a large circle of friends. He was a son of the late Dr. Mercy B. Jackson, a Professor in the Boston University School of Medicine, and one of the first women to practice Homeopathy. She believed in Homeopathy as given us by Samuel Hahnemann, and named her son for him. Dr. Jackson, true to his name, during the many years of his practice devoted himself to Homeopathy in all its purity.

A wife and one son, aged thirteen, survive him.

JOHN STORER, M. D.

